# Investigation of the shortcomings of the consort 2010 statement for the reporting of group sequential randomised controlled trials

**DOI:** 10.1186/1745-6215-16-S2-O53

**Published:** 2015-11-16

**Authors:** Munyaradzi Dimairo, Abigail Stevely, Susan Todd, Steven Julious, Jonathan Nicholl, Daniel Hind, Cindy Cooper

**Affiliations:** 1 University of Sheffield, School of Health and Related Research, Sheffield, UK; 2 University of Sheffield, The Medical School, Sheffield, UK; 3 University of Reading, Department of Mathematics and Statistics, Reading, UK

## Background

It could be argued that adaptive designs are underused in clinical trials research. However, of the adaptive trials that have been undertaken there are concerns which could be linked to inadequate reporting of trial conduct. We examined compliance in the reporting of group sequential randomised controlled trials (RCTs) to investigate the issues.

## Methods

We undertook a systematic review by searching Ovid MEDLINE (01/01/2001 to 23/09/2014), supplemented with RCTs from an audit study. We included parallel group, confirmatory, group sequential RCTs that were prospectively designed using a Frequentist approach. Eligible RCTs were examined for reporting compliance against the CONSORT 2010 checklist. Proposed modifications were added to capture group sequential aspects such as use statistical bias correction due to early stopping.

## Results

24% (68/284) RCTs were eligible; most were published in high impact journals. We found that 46(68%) were stopped early, predominantly either for futility or efficacy. Group sequential aspects were largely inadequately reported. Only 7% (3/46) RCTs which stopped early reported use of statistical bias correction. 52(76%) RCTs failed to disclose methods used to minimise the risk of operational bias through the knowledge or leaking of interim results. Suboptimal compliance was found in items relating to: access to trial protocols: randomisation methods; details of randomisation concealment, and its implementation. Changes to trial methods and outcomes could not be determined in most trials, due to inaccessible protocols and amendments.

## Conclusions

Suboptimal reporting may lead to researchers questioning the robustness of trial conclusions of RCTs that stop early to change practice. Modification of the CONSORT statement to incorporate adaptive designs could be helpful. Assurance of scientific rigour through transparent and adequate reporting is paramount to the acceptability of findings from adaptive RCTs.

